# Effect of Interaction between Mealworm Protein and Myofibrillar Protein on the Rheological Properties and Thermal Stability of the Prepared Emulsion Systems

**DOI:** 10.3390/foods9101443

**Published:** 2020-10-12

**Authors:** Tae-Kyung Kim, Min Hyeock Lee, Hae In Yong, Samooel Jung, Hyun-Dong Paik, Hae Won Jang, Yun-Sang Choi

**Affiliations:** 1 Research Group of Food Processing, Korea Food Research Institute, Wanju 55365, Korea; privacykin@naver.com (T.-K.K.); mhlee@kfri.re.kr (M.H.L.); awsm_y@kfri.re.kr (H.I.Y.); 2 Division of Animal and Dairy Science, Chungnam National University, Daejeon 34134, Korea; samooel@cnu.ac.kr; 3 Department of Food Science and Biotechnology of Animal Resources, Konkuk University, Seoul 05029, Korea; hdpaik@konkuk.ac.kr

**Keywords:** edible insect, alternative protein, food quality, calorimetry, viscosity

## Abstract

In this study, we investigated the effect of replacing myofibrillar protein (pork ham) with edible insect proteins (*Tenebrio molitor* L.) in meat emulsion systems and examined the interaction between the two types of proteins. We also evaluated the rheological properties and thermal stability of these meat emulsions. The replacement ratios of myofibrillar protein and edible insect protein were as follows: 100:0 (EI0), 80:20 (EI20), 60:40 (EI40), 40:60 (EI60), 20:80 (EI80), and 0:100 (EI100). The pH, redness, and yellowness of the emulsion systems, after replacing myofibrillar protein with *T. molitor* protein, significantly increased with *T. molitor* protein concentrations. In contrast, the lightness, hardness, cohesiveness, gumminess, chewiness, apparent viscosity, and differential scanning calorimetry (DSC) of the emulsion systems decreased significantly with increasing *T. molitor* protein concentrations. The backscattering values of EI0, EI20, and EI40 decreased evenly in all spots of the dispersions as the storage time increased. Thus, up to 40% of pork myofibrillar protein could be replaced with *T. molitor* protein in meat emulsion systems. The results also suggest that the interaction between edible insect protein and myofibrillar protein degrades the rheological properties and thermal stability of the meat emulsion systems.

## 1. Introduction

In recent studies, edible insects have been highlighted as an important protein resource for the future, in terms of food security [1,2]. Furthermore, edible insects can drastically reduce the production of greenhouse gases, such as carbon dioxide and methane, as compared to traditional protein sources, such as cow, pig, and chicken [3]. Edible insects are attracting attention as a future food because they have a short life cycle and low space requirement [4,5]. Although an increasing wiliness to eat insects has been recently reported from the several Western countries, a majority of customers remain reluctant to consume edible insects due to their gross appearance [6,7]. According to the sources, the hidden consumption of dried, ground insects in familiar products, such as pasta or ham, might lead to decreasing the neophobia and promote insects as food [7,8]. Therefore, research on protein extract should be conducted to expand the possibilities of various forms and applications of edible insects.

Myofibrillar protein is a major component of muscle protein and has a significant effect on the processing and manufacturing of meat products [9]. Myofibrillar protein is a salt-soluble protein that is known to denature during thermal processing to form a gel [10]. The protein gel consists of a stable three-dimensional network structure, which forms due to the polymerization reaction between the protein molecules [11]. The formation of these protein gels is significant as they contribute to water entrapment, meat binding, and fat immobilization in protein resources of food products [12]. Furthermore, a myofibrillar protein network and fat are important components that affect the textural properties of meat emulsion [10]. Fat plays a significant role in the improvement of texture of meat emulsion [13,14]. Fat is dispersed to be further stabilized through hydrophobic interactions and disulfide linkages with the myofibrillar protein membrane during the emulsifying process [12]. Emulsified fats have a structure that results in the formation of globule-like copolymers, which occupy the empty space inside the gel networks, making it more compact [15]. In emulsion systems, the emulsification of myofibrillar protein and fat is important and a vital factor in all meat emulsion products [16]. However, myofibrillar protein is mainly derived from livestock, and livestock breeding is restricted due to social changes such as environmental pollution. Thus, it is necessary to save myofibrillar protein derived from livestock. Edible insects are emerging as a valuable source of protein. The key to this study is to determine the amount of edible insect protein that can be used as an alternative to myofibrillar protein.

The objective of this study was to examine the thermal stability and rheological properties of emulsion systems prepared by replacing myofibrillar protein with proteins obtained from the edible insect, *Tenebrio molitor* L., and to contribute to the development of an interaction between edible insect protein and myofibrillar protein.

## 2. Materials and Methods

### 2.1. Protein Extraction from Edible Insect

The freeze-dried *T. molitor* was obtained from a local market (Farm bang, Sunchang, Korea). The insects were grown in cemented boxes (with fermented saw dust on the floor) for 3 months. The feed withdrawal was done for 2 days before freezing (−70 °C). The freeze-dried edible insects were pulverized with a blender and then filtered through a 535-μm pore size steel sieve. One part of the ground *T. molitor* sample was treated with one part 0.58 M saline (0.49 M NaCl, 17.8 mM Na_5_P_3_O_10_, and 1 mM NaN_3_, pH 8.3, 2 °C). The solution was homogenized for 30 s and then incubated overnight at 4 °C. The slurry was incubated at 4 °C for 1 h and then centrifuged (18,000× *g*, 2 °C) for 30 min (Supra 25K High speed refrigerated centrifuge; Hanil Science Industrial, Seoul, Korea). The protein extract was sieved through three layers of cheesecloth. The protein concentration of *T. molitor* extract was adjusted to 30 mg/mL.

### 2.2. Myofibrillar Protein Extraction from Pork Ham

The myofibrillar protein extraction was performed using the method described by Choi et al. [10]. Briefly, fresh pork lean meat was ground through a 3-mm plate. One part of the ground meat and two parts of 0.58 M saline solution were homogenized for 30 s. The slurry was then centrifuged at 18,000× *g* at 4 °C for 30 h. The myofibrillar protein extract was finally strained through layers of cheesecloth. The protein concentration of myofibrillar protein extract was adjusted to 30 mg/mL.

### 2.3. Preparation of Emulsion Systems

The emulsion systems were prepared according to the replacement ratio of myofibrillar protein to edible insect protein. The ratios of myofibrillar protein and edible insect protein were as follows; 100:0 (EI0), 80:20 (EI20), 60:40 (EI40), 40:60 (EI60), 20:80 (EI80), and 0:100 (EI100). Pork back fats were extracted and purified according to a previously described method [17]. Mixed protein solution and fat (in a 4:1 *v*/*w* ratio) were homogenized at 14,500 rpm for 60 s using a high-speed homogenizer (T-25; Janke & Kunkel, Staufen, Germany). The temperature of the emulsion systems was monitored and maintained below 5 °C. The preparation of all emulsion systems and further experiments were conducted on the same day. 

### 2.4. pH

The pH values of the emulsion system samples were measured with a pH meter after homogenizing the sample (5 g) and distilled water (20 mL).

### 2.5. Color Evaluation

The color (CIE L*, a*, and b* values) of the emulsion systems were determined with a colorimeter (CR-410; Minolta Co., Osaka, Japan; illuminate C). The colorimeter was calibrated with a white plate (L* = +97.83, a* = −0.43, b* = +1.98). 

### 2.6. Water Holding Capacity 

The water holding capacity (WHC) of the emulsion systems was measured using the gravimetric methods of Kocher and Foegeding [18]. Briefly, the emulsion systems were heated and centrifuged at 1,000× *g* for 10 min (4 °C). WHC was measured by calculating the released moisture content. 

### 2.7. Rheological Properties

The apparent viscosity of the emulsion systems was determined with a Brookfield viscometer (DV3T HB; Brookfield Engineering Labs, Inc., Stoughton, MA, USA). The viscometer was attached to a standard cylinder sensor (SC4-29). The standard cylinder was filled with the emulsion systems and was spun at a set shear rate (s^−1^). Dynamic viscosity was measured as a function of frequency using a Physica MCR 101 Rheometer (Anton Paar Ltd., Graz, Austria), and measurements were obtained using a parallel plate with a diameter of 50 mm and a gap of 1 mm. The angular frequency ranged from 0.1 to 100 rad/s at a strain of 0.5%, and the temperature was maintained at 25 °C. Storage modulus (G′) and loss modulus (G″) were recorded using RheoCompass v. 1.19 software.

### 2.8. Emulsion Stability

The emulsion stability of the emulsion systems was quantified using a Turbiscan Tower (Formulaction, L’Union, France). Briefly, the emulsion samples were transferred to transparent containers, and the transmittance and backscattering of a pulsed near-infrared light (λ = 880 nm) by the sample were measured from the bottom to the top at 40-μm intervals. The measurements were taken at intervals of 1 min for a total of 3 h at 25 °C.

### 2.9. Differential Scanning Calorimetry 

The emulsion thermal stability was tested with differential scanning calorimetry (DSC) 4000 (PerkinElmer, Waltham, MA, USA). An empty aluminum pan (used as a reference) was placed on one side and a sealed pan (containing 20 mg of sample) was placed on the other side to detect the change in calories. The initial temperature was set at 20 °C and held for 2 min before heating. The test temperature range was 20–120 °C, and the temperature was increased at the rate of 10 °C/min [19]. 

### 2.10. Texture Profile Analysis

The texture profile analysis (TPA) of meat emulsion systems was determined with a texture analyzer (TA-XTplus; Stable Micro Systems, Ltd., Surrey, England, UK). The emulsion system was cooled (24 °C) after the thermal processing step. The heated samples were procured from the central portion of each system [20].

### 2.11. Sodium Dodecyl Sulfate-Polyacrylamide Gel Electrophoresis 

Sodium dodecyl sulfate-polyacrylamide gel electrophoresis (SDS-PAGE) was performed to determine the distribution of proteins based on molecular weight. All chemicals were obtained from Bio-Rad Laboratories, Inc. (Hercules, CA, USA). A 20 μg protein sample was mixed with buffer solution and heated for 5 min at 100 °C. The cooled sample was added to the wells of 4–20% Mini-PROTEIN^®^ TGX and loaded at 80 mA with the standard marker. After separation, the gels were stained using 0.25% Coomassie brilliant blue.

### 2.12. Statistical Analysis

All experimental data were evaluated using SPSS v. 20.0 (SPSS, Inc., Chicago, IL, USA) in a completely randomized study design. One-way analysis of variance was used to determine significant (*p* < 0.05) differences among the treatment groups, and Duncan’s multiple range test was applied to analyze differences in the rheological properties and thermal stability of the emulsion systems. 

## 3. Results and Discussions

### 3.1. pH, Color, and Water Holding Capacity 

Table 1 shows the pH, color, and WHC of the emulsion systems prepared with the interaction between edible insect protein and myofibrillar protein. The pH of the emulsion systems after replacing myofibrillar protein with *T. molitor* protein was significantly higher (*p* < 0.05), and it increased with *T. molitor* protein concentrations. These results are in agreement with those of Choi et al. [8] who reported that replacing pork meat with yellow mealworm increases the pH of frankfurters. Similarly, Kim et al. [3] reported that the pH of emulsion sausages with mealworm larvae was higher than control without mealworm larvae. This could explain the higher pH of *T. molitor* protein as compared to myofibrillar protein. According to Kim et al. [21], the evaluation of pH of edible insect proteins could provide insights into protein functionality-linked facts. The lightness of the emulsion systems prepared with *T. molitor* protein reduced significantly (*p* < 0.05) with increasing *T. molitor* protein concentrations. In addition, the redness and yellowness of the emulsion systems increased significantly (*p* < 0.05) with increasing *T. molitor* protein concentrations. These results are consistent with those by Choi et al. [8] who observed that replacing pork meat with yellow mealworm results in lower lightness and higher yellowness of frankfurters. Smarzyński et al. [22] reported that the color of the product is known to play very important role in the acceptance of customers, and then observed that the respondents dislike the product containing higher concentrations of cricket flour. Kim et al. [3] noted that the color of emulsion sausages pre-treated with edible insects is darker and yellower than that of the control. Generally, edible insect protein is brown or dark in color, which may be attributed to the melanin pigments and extraction process of the edible insects [23,24]. Thus, the color of the emulsion systems formulated by replacing myofibrillar protein with *T. molitor* protein may be affected by protein aggregation and color pigment oxidation. The WHC of the emulsion systems prepared by substituting myofibrillar protein with *T. molitor* protein reduced significantly (*p* < 0.05) with increasing *T. molitor* protein concentrations. This could be explained by the fact that myofibrillar protein has a more stable structure than edible insect protein, so it has better efficiency in trapping water molecules [10,25]. 

### 3.2. Rheological Properties 

**Figure 1:**
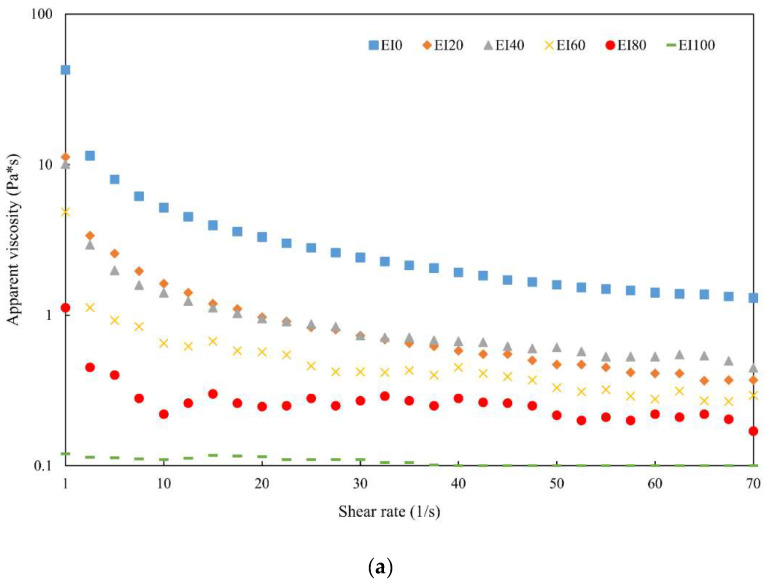

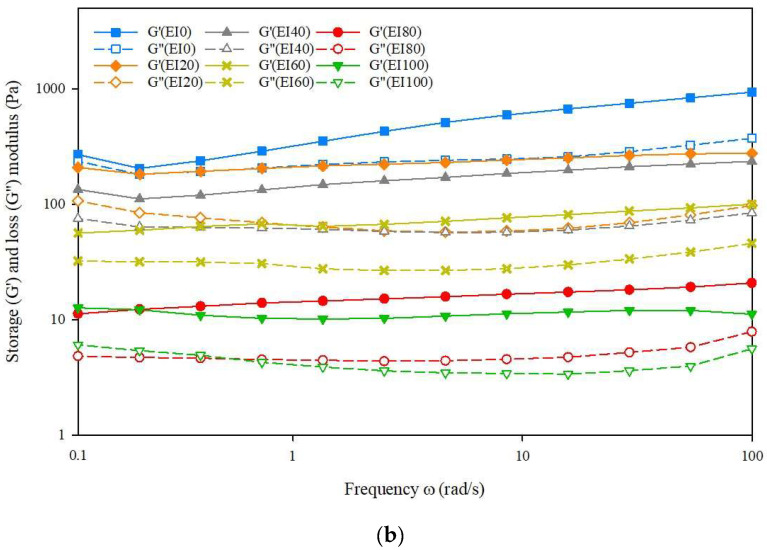
Rheological properties (apparent viscosity (**a**), dynamic viscosity (**b**)) of emulsion prepared with the interaction between porcine myofibrillar protein and extracted edible insect protein. Porcine myofibrillar protein was substituted by edible insect protein at 0, 20, 40, 60, 80, and 100% (EI0, EI20, EI40, EI60, EI80, and EI100, respectively).

The interaction between myofibrillar protein and *T. molitor* protein significantly affected the apparent viscosity of the emulsion systems (Figure 1a). The apparent viscosity of the emulsion systems prepared by replacing myofibrillar protein with *T. molitor* protein decreased significantly (*p* < 0.05) with increasing *T. molitor* protein concentrations. These results are in line with those described by Choi et al. [8] who showed that the apparent viscosity of the control was higher than that of all treatments with yellow mealworm, and replacing pork meat with increasing concentrations of yellow mealworm resulted in lower apparent viscosity. These results may be explained by the reduced water binding and fat binding capacities of edible insect protein [3,8]. The behavior of the dynamic rheology of emulsion prepared with the interaction between porcine myofibrillar protein and extracted edible insect protein as a function of frequency is depicted in Figure 1b. The G′ and G″ represent the energy stored in the elastic structure and the energy lost by viscous dissipation per cycle of deformation, respectively [26]. Similar to the results of apparent viscosity, the values of G′ and G″ decreased with increasing *T. molitor* protein concentrations. In addition, there was no crossover of the values of G′ and G″ with the increase in the measured angular frequency range for all samples. In the case of emulsion stabilized with proteins such as myofibril, the value of G′ was generally higher than that of G″, thus indicating a more stable emulsion system due to the restricted structure [27].

### 3.3. Emulsion Stability

**Figure 2:**
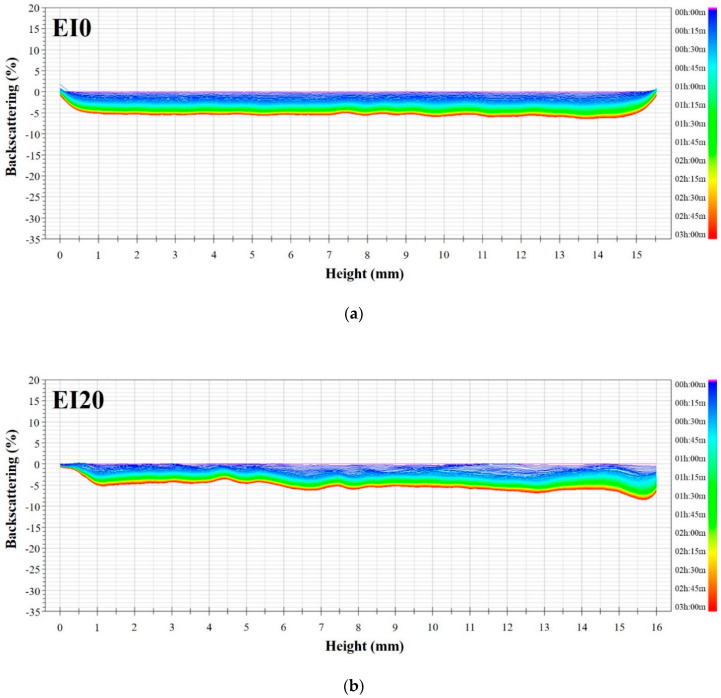

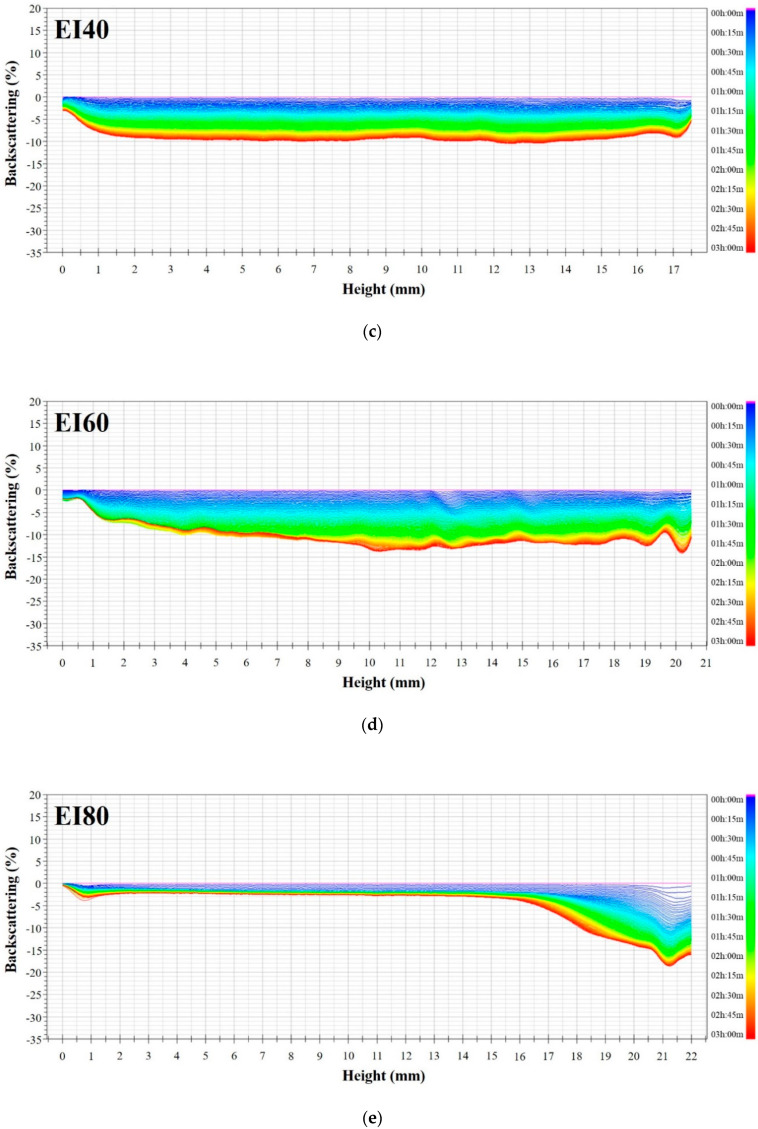

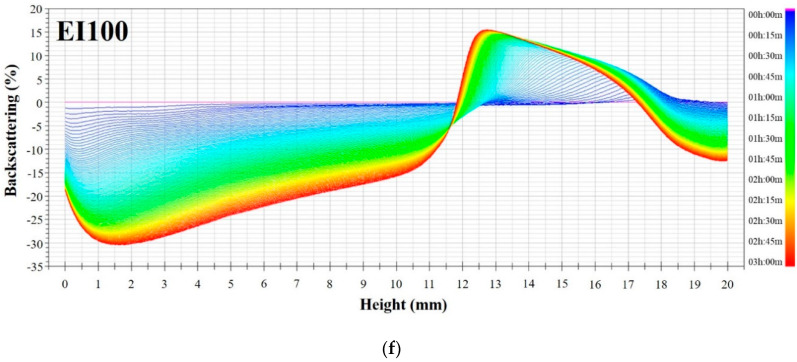
Backscattering profiles of emulsion prepared with interaction between porcine myofibrillar protein and extracted edible insect protein: (**a**) EI0, (**b**) EI20, (**c**) EI40, (**d**) EI60, (**e**) EI80, and (**f**) EI100. Porcine myofibrillar protein was substituted by edible insect protein at 0, 20, 40, 60, 80, and 100% (EI0, EI20, EI40, EI60, EI80, and EI100, respectively).

The stability of emulsion systems is a major factor when characterizing the quality of emulsified meat products. In general, during homogenization of immiscible oil and water, proteins reduce the interfacial tension and form protective layers around oil droplets, thereby providing stability against droplet coalescence [28]. In addition, emulsion formation and stability could be impacted by the content and size, amino acid composition, hydrophobicity, hydrophilicity, and conformational characteristics of the proteins [29]. Figure 2 shows backscattering profiles of the interaction between porcine myofibrillar protein and extracted edible insect protein. As shown in Figure 2a–d, the overall backscattering values decreased during storage. Especially, the backscattering values of EI0, EI20, and EI40 decreased evenly in all spots of the dispersions as the storage time increased. In case of micro-emulsions, with droplet size larger than 600 nm, the reduction in backscattering values during storage implied that the increment in the droplet size was caused by emulsion destabilization such as flocculation and coalescence. Likewise, the backscattering values of EI60 and EI80 decreased as the storage time increased (Figure 2d,e). However, the variations in backscattering reduction between the dispersion layers were observed. Especially, in the case of EI80, there were a few reductions in backscattering of bottom and middle layers, whereas it was distinct at the top layer of the dispersion. This phenomenon could be attributed to the emulsion destabilization due to creaming. As the myofibrillar proteins required for the emulsification of fat droplets were not enough, the fat molecules remained afloat. Myofibril protein can be absorbed at the interface between water and fat droplets during the formation of the emulsion, which contributes to the prevention of gravitational separation, flocculation, and coalescence due to its hydrophobic amino acid residue such as SH group [27,30]. Conversely, the backscattering profile of EI100 showed a considerable difference when compared to others (Figure 2f). The backscattering value of EI100 decreased at the bottom and top layers, whereas the value increased at the middle layer during storage. Similar to EI80, the reduction in backscattering value at the top layer indicated cream formation of the fat droplets. However, the rapid reduction in backscattering value at the bottom layer could be explained by the sedimentation and packing of protein molecules, which are not suitable for emulsifying fat droplets. As a result, the middle layer of the dispersion became clear by floatation of fat droplets and sedimentation of proteins, which resulted in the increase of backscattering value at the middle layer. According to the result from SDS-PAGE (Section 3.6), the protein extracted from edible insect was relatively smaller than the myofibrillar protein from pork ham. The size of the proteins is also one of the most important factors that affects emulsion formation and stability. Although proteins with small molecular weight display good emulsion formation abilities due to rapid diffusion to the interface between water and fat droplet, they have low emulsion stability. Proteins with large molecular weight could improve emulsion stability once the layer around the fat and water interface has formed [29]. In addition, higher viscosity due to proteins with a large molecular weight can help stabilize the emulsion system. Based on these results, protein from edible insects could be used as a substitute for myofibrillar protein from pork to some extent for manufacturing meat products requiring an emulsion system.

### 3.4. Differential Scanning Calorimetry 

**Figure 3:**
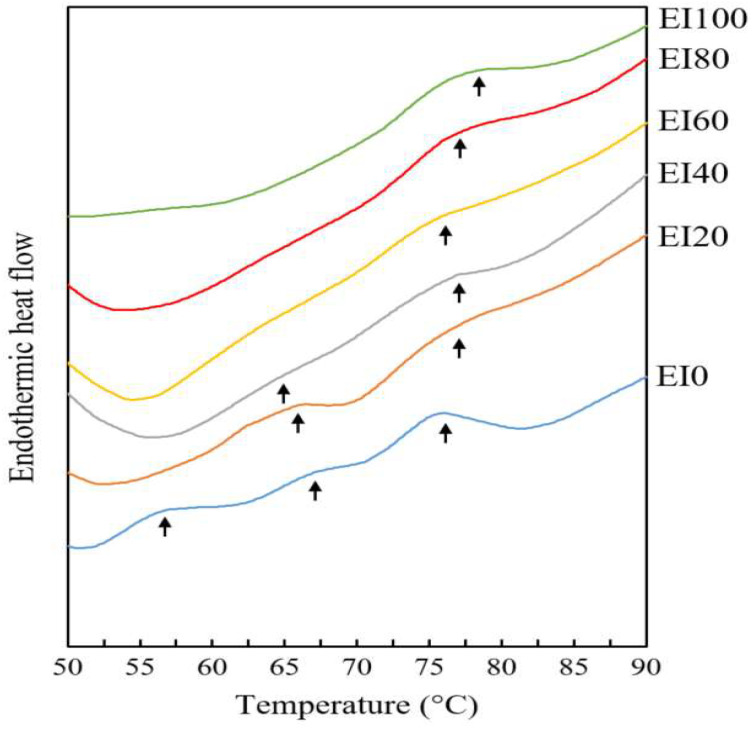
Endothermic heat flow of emulsion prepared with the interaction between porcine myofibrillar protein and extracted edible insect protein. Porcine myofibrillar protein was substituted by edible insect protein at 0, 20, 40, 60, 80, and 100% (EI0, EI20, EI40, EI60, EI80, and EI100, respectively).

A differential scanning calorimeter is used to detect the heat flow changes in protein, which might be due to protein denaturation or aggregation induced by thermal energy [31]. When the endothermic heat flow of EI0 (which was composed of only porcine myofibrillar protein) was examined, different peak temperatures were detected at 57.07, 67.24, and 76.30 °C. Myosin, sarcoplasmic, and actin are generally denatured around 40–60, 6–70, and 71–83 °C, respectively, and the peak temperatures of EI0 were detected in this temperature range. However, these peaks disappeared when edible insect protein was added (Figure 3). The peak point between 55 and 60 °C was not detected in the emulsion systems in which edible insect protein was added, and the peak point between 60 and 70 °C was not observed after substituting myofibrillar protein with edible insect protein by more than 60%. According to Lee et al. [32], aggregation of extracted protein from mealworm increases significantly above 75 °C and below 95 °C. Results from our study showed similar protein denaturation temperature above 75 °C. Pork myofibrillar protein is composed of typical protein (sarcoplasmic, actin, and myosin). However, the whole form of *T. molitor* was used to extract protein and various complex components such as hemolymph, skeletal muscle, enzyme, chitin, and others were also extracted, and this complex protein extract had a higher heat stability than that of pork myofibrillar protein [23].

### 3.5. Texture Profile Analysis

The effects of the interaction between *T. molitor* protein and myofibrillar protein on the textural profiles of the emulsion systems are shown in Table 2. Replacement of myofibrillar protein with increasing concentrations of *T. molitor* protein led to significant reduction in the hardness of the emulsion systems (*p* < 0.05), and the emulsion system with myofibrillar protein (EI0) had the highest (*p* < 0.05) hardness. The springiness of the emulsion systems with myofibrillar protein (EI0) was the highest (*p* < 0.05), whereas the springiness of the emulsion systems (EI100) with *T. molitor* protein was the lowest (*p* < 0.05). The cohesiveness of the emulsion systems with *T. molitor* protein was the lowest (*p* < 0.05), and the cohesiveness of emulsion systems prepared with myofibrillar protein ranged from 0.47 to 0.53. The gumminess and chewiness of the emulsion systems prepared by replacing myofibrillar protein with *T. molitor* protein was lower (*p* < 0.05). Similar results have been reported by Choi et al. [8] who found that the hardness, gumminess, and chewiness of the control (with only pork ham) were the highest, and the hardness of frankfurters with yellow mealworm decreased as the concentration of yellow mealworm was increased up to 20%. Kim et al. [3] reported that pre-treated mealworm larvae and silkworm pupae, as a protein ingredient in emulsion sausage, and all emulsion sausages with pre-treated edible insect was higher than that of the control (without pre-treated edible insect). Park et al. [33] reported that the hardness, gumminess, and chewiness of meat batter prepared with edible silkworm pupae increased with increase in edible silkworm pupae concentration. These results indicated an improvement in textural properties when edible silkworm was added to the meat batter. Although several studies have investigated edible insect protein as an additive to meat products, studies on replacement of meat are lacking. In this study, the interaction between the myofibrillar protein and the edible insect protein appeared to be weak, and it seems complete replacement of myofibrillar protein with edible insect protein was difficult. Thus, in order to replace meat with edible insects, it is important that the textural properties of the emulsion systems are retained by applying various technologies.

### 3.6. SDS-Polyacrylamide Gel Electrophoresis

**Figure 4:**
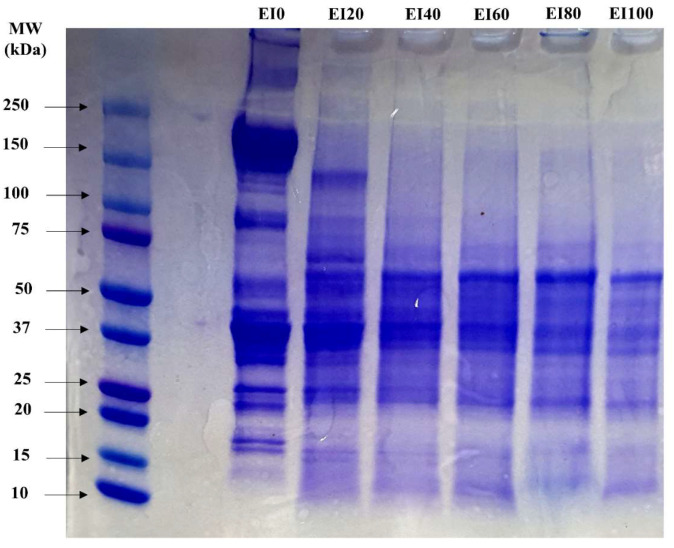
Molecular weight distribution of emulsion prepared with the interaction between porcine myofibrillar protein and extracted edible insect protein. Porcine myofibrillar protein was substituted by edible insect protein at 0, 20, 40, 60, 80, and 100% (EI0, EI20, EI40, EI60, EI80, and EI100, respectively).

The molecular weight of a protein is dependent on its hydrophobic residue and/or structure, and based on these characteristics, SDS-PAGE is used to estimate the protein type [34]. Obvious protein bands of myosin heavy chain and actin were observed in EI0 [10]. However, an increased substitution ratio in *T. molitor* protein changed the protein distribution (Figure 4). This result showed that, even in the case of *T. molitor* protein, which was extracted using 0.58 M saline solution, muscular protein could not be extracted easily. Furthermore, after the addition of insect protein, high molecular weight bands (above 50 kDa) were faint, while bands under 10 kDa were not observed in E10. This could be due to the fact that the most abundant proteins in *T. molitor* are hemolymph (under 12 kDa), alpha-amylase (under 50 kDa), and actin (30–50 kDa) [35]. Therefore, protein distribution tended to large amount of lower molecular weight [36,37].

## 4. Conclusions

The replacement of myofibrillar protein with *T. molitor* protein significantly reduced the thermal stability and rheological properties of the emulsion systems and contributed to the development of an interaction between edible insect protein and myofibrillar protein. Based on the results of this study, it can be concluded that about 40% of pork myofibrillar protein can be replaced with edible insect proteins. In addition, the results suggest that the interaction between edible insect protein and myofibrillar protein may degrade the quality of the emulsion system.

## Figures and Tables

**Table 1:** pH, color, and water holding capacity (WHC) of the emulsion prepared with porcine myofibrillar protein and extracted edible insect protein.

	EI0	EI20	EI40	EI60	EI80	EI100
pH	6.29 ± 0.02 ^e^	6.32 ± 0.01 ^d^	6.38 ± 0.02 ^c^	6.39 ± 0.01 ^bc^	6.41 ± 0.01 ^ab^	6.43 ± 0.02 ^a^
CIE ^(1)^ L*	73.76 ± 0.08 ^a^	65.08 ± 0.15 ^b^	59.75 ± 0.18 ^c^	55.25 ± 0.17 ^d^	51.12 ± 0.21 ^e^	48.84 ± 0.02 ^f^
CIE a*	−0.13 ± 0.03 ^f^	0.73 ± 0.03 ^e^	1.82 ± 0.03 ^d^	2.37 ± 0.02 ^c^	2.52 ± 0.01 ^b^	2.85 ± 0.02 ^a^
CIE b*	9.15 ± 0.06 ^f^	9.92 ± 0.10 ^e^	10.11 ± 0.05 ^d^	10.63 ± 0.12 ^c^	10.78 ± 0.09 ^b^	11.19 ± 0.01 ^a^
WHC (%)	53.32 ± 3.42 ^a^	18.69 ± 5.28 ^bc^	22.53 ± 2.58 ^b^	16.01 ± 1.78 ^d^	16.45 ± 3.55 ^d^	9.82 ± 0.39 ^e^

All values are mean ± standard deviation of three replicates (*n* = 3). a–f Means within a row with different letters are significantly different (*p* < 0.05). Porcine myofibrillar protein was substituted by edible insect protein at 0, 20, 40, 60, 80, and 100% (EI0, EI20, EI40, EI60, EI80, and EI100, respectively). ^(1)^ CIE, International Commission on Illumination.

**Table 2:** Texture profile analysis of emulsion prepared with the interaction between porcine myofibrillar protein and extracted edible insect protein.

	EI0	EI20	EI40	EI60	EI80	EI100
Hardness (kg)	0.60 ± 0.06 ^a^	0.22 ± 0.03 ^b^	0.15 ± 0.01 ^c^	0.08 ± 0.01 ^d^	0.06 ± 0.01 ^d^	0.05 ± 0.02 ^d^
Springiness	0.99 ± 0.01 ^a^	0.97 ± 0.01 ^b^	0.97 ± 0.01 ^b^	0.97 ± 0.01 ^b^	0.97 ± 0.01 ^b^	0.86 ± 0.11 ^b^
Cohesiveness	0.47 ± 0.05 ^a^	0.47 ± 0.03 ^a^	0.53 ± 0.03 ^a^	0.51 ± 0.03 ^a^	0.47 ± 0.07 ^a^	0.29 ± 0.22 ^b^
Gumminess (kg)	0.28 ± 0.04 ^a^	0.10 ± 0.01 ^b^	0.08 ± 0.01 ^c^	0.04 ± 0.01 ^d^	0.03 ± 0.01 ^de^	0.01 ± 0.01 ^e^
Chewiness (kg)	0.28 ± 0.04 ^a^	0.10 ± 0.01 ^b^	0.08 ± 0.01 ^c^	0.04 ± 0.01 ^d^	0.03 ± 0.01 ^de^	0.01 ± 0.01 ^e^

All values are mean ± standard deviation of three replicates (*n* = 3). a–e Means within a row with different letters are significantly different (*p* < 0.05). Porcine myofibrillar protein was substituted by edible insect protein at 0, 20, 40, 60, 80, and 100% (EI0, EI20, EI40, EI60, EI80, and EI100, respectively).
